# *MAL73*, a novel regulator of maltose fermentation, is functionally impaired by single nucleotide polymorphism in *sake* brewing yeast

**DOI:** 10.1371/journal.pone.0198744

**Published:** 2018-06-12

**Authors:** Takumi Ohdate, Fumihiko Omura, Haruyo Hatanaka, Yan Zhou, Masami Takagi, Tetsuya Goshima, Takeshi Akao, Eiichiro Ono

**Affiliations:** 1 Research Institute, Suntory Global Innovation Center (SIC) Ltd., Seika-cho, Soraku-gun, Kyoto, Japan; 2 National Research Institute of Brewing, Higashihiroshima, Hiroshima, Japan; CNR, ITALY

## Abstract

For maltose fermentation, budding yeast *Saccharomyces cerevisiae* operates a mechanism that involves transporters (*MALT*), maltases (*MALS*) and regulators (*MALR*) collectively known as *MAL* genes. However, functional relevance of *MAL* genes during *sake* brewing process remains largely elusive, since *sake* yeast is cultured under glucose-rich condition achieved by the co-culture partner *Aspergillus* spp.. Here we isolated an ethyl methane sulfonate (EMS)-mutagenized *sake* yeast strain exhibiting enhanced maltose fermentation compared to the parental strain. The mutant carried a single nucleotide insertion that leads to the extension of the C-terminal region of a previously uncharacterized *MALR* gene *YPR196W-2*, which was renamed as *MAL73*. Introduction of the mutant allele *MAL73L* with extended C-terminal region into the parental or other *sake* yeast strains enhanced the growth rate when fed with maltose as the sole carbon source. In contrast, disruption of endogenous *MAL73* in the *sake* yeasts decreased the maltose fermentation ability of *sake* yeast, confirming that the original *MAL73* functions as a MALR. Importantly, the *MAL73L-*expressing strain fermented more maltose in practical condition compared to the parental strain during *sake* brewing process. Our data show that *MAL73*(*L*) is a novel *MALR* gene that regulates maltose fermentation, and has been functionally attenuated in *sake* yeast by single nucleotide deletion during breeding history. Since the *MAL73L-*expressing strain showed enhanced ability of maltose fermentation, *MAL73L* might also be a valuable tool for enhancing maltose fermentation in yeast in general.

## Introduction

Generally, yeast alters its gene expression pattern in response to environmental nutritional conditions, notably including sugar and nitrogen sources. Alteration of gene expression in response to environmental sugar availability has been extensively studied using the budding yeast *Saccharomyces cerevisiae* [1]. Maltose, which is a α-disaccharide comprising two α-glucose monomers, is present at high levels in malt prepared from germinated barley (*Hordeum vulgare*) seed. Maltose fermentation in *S*. *cerevisiae* requires a set of *MAL* genes (*i*.*e*. genes encoding a maltose transporter (MALT), maltase (MALS), and zinc finger-type transcription factors (MALR) that positively regulate *MALT* and *MALS* genes [2]. To date, Gibson et al. [3] have reported the sequence of several *MALR* genes (*MAL23*, *MAL43*, and *MAL63*), and Hu et al. [4] have defined the functional domains of MALR proteins. On the other hand, *MAL13* and *MAL33* do not seem to be functionally relevant in yeast under laboratory conditions [5, 6]. These *MAL* genes are crucial for fermentation of maltose, as demonstrated by the observation that yeasts lacking any of these genes fail to grow in medium in which maltose is the sole carbon source [7]. Five structurally similar, but genetically unlinked, *MAL* loci (*MAL1* to *MAL4* and *MAL6*) have been shown to each contain up to three different genes, including: a *MALx1* (where *x* = 1 to 4 or 6) gene encoding a maltose transporter (MALT); a *MALx2* gene, encoding a maltase protein (MALS); and a *MALx3* gene, a transcriptional regulator (MALR) of other *MAL* genes [7–11]. These *MAL* loci are located proximal to the telomeric region of the respective chromosome (*MAL1* on chromosome VII, *MAL2* on chromosome III, *MAL3* on chromosome II, *MAL4* on chromosome XI, and *MAL6* on chromosome VIII). In medium containing high concentrations of glucose, glucose uptake is promoted via hexose transporters (HXTs) and maltose uptake is simultaneously transcriptionally repressed, a cellular response that is known as a part of the “glucose repression” phenomenon [12]. For example, the maltose transporter-encoding *MAL11* gene is repressed in the presence of glucose [13].

In some yeast strains, maltose fermentation ability has been compromised or completely abolished during their evolution or via selection during domestication, as demonstrated by the loss-of-function mutation in, or absence of, one or more *MAL* loci. The molecular mechanism(s) regulating maltose fermentation have yet to be elucidated in some commercially important yeast strains such as Japanese *sake* yeast [14]. Among brewing yeasts, beer yeasts show high maltose fermentation ability, including the diversification of various kinds of *MAL* genes, presumably due to artificial selection under conditions favoring malt-based fermentation. In contrast, Japanese *sake* yeast strains, which are utilized for fermentation in the glucose-rich environments resulting from the activity of fungal (*Aspergillus oryzae*) glucoamylases, show only a weak maltose fermentation ability [15]. Although the genome sequence of the Japanese *sake* yeast K7 strain, which is a major *sake* yeast, has been reported [16], the basis for the weak maltose fermentation ability of *sake* yeasts remains elusive. In the present study, we focused on the low maltose fermentation ability of *sake* yeast, and employed a genetic approach to enhance this property. Here we show that a putative *MALR* gene *YPR196W-2*, renamed as *MAL73*, encodes a novel hypomorphic regulatory protein whose function can be enhanced by a single nucleotide insertion that leads to the expression of *MAL73L* harboring an extended C-terminal region. In turn, the data also suggest that *MAL73L* has been functionally attenuated in *sake* yeast through frameshifts during breeding history. *MAL73L* expression in the original *sake* yeast strain resulted in enhanced growth on maltose medium compared to the parental strain. Moreover, *MAL73L-*expressing strain was capable of fermenting *sake* slightly faster than a control yeast strain. These results suggest that *MAL73L* is a genetic tool valuable for improving the maltose fermentation ability of yeast.

## Materials and methods

### Strains and media

Yeast strains used in this study are X2180-1A (MAT a, ATCC26786) and Kyokai No. 7 (K7) series strains (K7, K9, K1001, K1601, and K1801; the Brewing Society of Japan). Cells were cultured in YPD (2% glucose, 1% yeast extract, and 2% peptone), YPM (2% maltose, 1% yeast extract, 2% peptone), SD medium (2% glucose, 0.67% yeast nitrogen base), SMal medium (2% maltose, 0.67% yeast nitrogen base), SD+Antimycin A (SD medium containing antimycin A; 0.67% yeast nitrogen base (Difco), 2% maltose, 2 mg/L antimycin A), or MA medium (SMal+antimycin A; SMal medium containing antimycin A; 0.67% yeast nitrogen base (Difco), 2% maltose, 2 mg/L antimycin A). Antimycin A is known to inhibit the mitochondrial electron transfer system, thus permitting stringent assessment of maltose fermentation ability [12].

### Gene disruption

Primers used in this study are summarized in Table 1. The *YPR196W-2*Δ mutant was generated by using the CRISPR/Cas9 system [17] as described previously in DiCarlo et al. [18]. Briefly, gRNA (guide RNA) was generated as follows. For the upstream gRNA, the 1st PCR was performed by using the primer pairs gRNA-F + YRP196W-2-N1 and YRP196W-2-N2 + gRNA-R. The two resulting PCR fragments were combined by using the GeneArt Seamless Cloning and Assembly Enzyme Mix (Thermo Fisher Scientific), yielding the desired gRNA. To amplify this gRNA, the 2nd PCR was performed by using the primer pair gRNA-F + gRNA-R with gRNA as a template. The resulting gRNA PCR fragment was introduced into the strain carrying Cas9 plasmid. To generate the downstream gRNA, the 1st PCR was performed by using the primer pairs gRNA-F + YRP196W-2-C1 and YRP196W-2-C2 + gRNA-R. The subsequent steps were as described above for the standard gRNA construction. A PCR fragment containing the hygromycin B resistance-encoding gene (*hygB*) was amplified by using the primer pair YRP196W-2-hyg-F + YRP196W-2-hyg-R with pYC240 plasmid as the template [19]. These three fragments (upstream gRNA, downstream gRNA, and the *hygB* fragment) were introduced into cells carrying Cas9 plasmid, and the resulting yeast transformants were selected using 100 μg/ml hygromycin B-containing YPD plates. After verification of the *YRP196W-2* gene disruption, transformants were grown in the absence of selection for the Cas9 plasmid, and loss of the plasmid was confirmed by screening.

**Table 1:** Primers used in this study.

Primer	Sequence
Cas9-F	5’-AATTAACTAAACAGGCCTATGGCTAGTATGCAGAAACTGA-3’
Cas9-R	5’-TAATTATTACTCAGGCCTTCACTTCTTCTTCTTTGCCTGT-3’
gRNA-F	5’-CCTTGGAGTAAAAATCAACTATCATC-3’
gRNA-R	5’-GTATCAGGCTAACGTGCACT-3’
MAL11-RT-F	5’-TTGGGTCATCATCGATCTGC-3’
MAL11-RT	5’-TCCGAATGGATCAACCACAG-3’
MAL33-RT-F	5’-AAGCGGTCCTAACACCATTG-3’
MAL33-RT-R	5’-ATAGAGCCGCAAGCACTGAT-3’
pYC-Z130-F	5’-CTCCACCTCAGCCAGAGTTC-3’
pYC-Z130-R	5’-GACTCCGTAGCCAAAGCATC-3’
TDH3pro-F	5’-CATTAATGCAGGTTGCGGCCGCACCAGTTCTCACACGGAAC-3’
TDH3pro-R	5’-AACCAAGGCCTGTTTATGTGTGTTTATTCGAA-3’
TDH3term-F	5’-TAAACAGGCCTTGGTTGAACACGTTGCC-3’
TDH3term-R	5’-CGGGCATTTAAATGCGGCCGCCC TCAATCAATGAATCGAAAATGTCA-3’
TDH3-RT-F	5’-CGGTAACATCATCCCATCC-3’
TDH3-RT-R	5’-CGGCAGCCTTAACAACCTTC-3’
TDH3-YPR196W-2-F	5’-ACACACATAAACAGGCCTATGACTTTGGCAAAACAGG-3’
TDH3-YPR196W-2-R	5’-CGTGTTCAACCAAGGCCTTAAGGAATTATGTCGTCTTCATC-3’
TEF2pro-F	5’-CATTAATGCAGGTTGCGGCCGCACTTACATATAGTAGATGTCAAGC-3’
TEF2pro-R	5’-TACTCAGGCCTGTTTAGTTAATTATAGTTCGTTGAC-3’
TEF2term-F	5’-TAAACAGGCCTGAGTAATAATTATTGCTTCCATATAATATT-3’
TEF2term-R	5’-CGGGCATTTAAATGCGGCCGCCCTACTGGGTAAATTTTGAAAG-3’
YPR196W-RT-F	5’-ATGTGATTGCTGTCGCGTTC-3’
YPR196W-RT-R	5’-GCTTTCTCCGATGGACTTGG-3’
YPR196W-2-N1	5’-GTCACACTTTACTCGACGAAGATCATTTATCTTTCACTGC-3’
YPR196W-2-N2	5’-TTCGTCGAGTAAAGTGTGACGTTTTAGAGCTAGAAATAGCAA-3’
YPR196W-2-C1	5’-TTGAAACATTTTATGATTGCGATCATTTATCTTTCACTGC-3’
YPR196W-2-C2	5’-GCAATCATAAAATGTTTCAAGTTTTAGAGCTAGAAATAGCAA-3’
YPR196W-2-hyg-F	5’-CGTTCCATTGTATTCAATAACTTAAACTAGTGAAGTTCCTATAC-3’
YPR196W-2-hyg-R	5’-CTCGCCTGAACATAGCTTCTTACACAGCGCTGAAGTTCCTATTC-3’
YPR196W-2-F	5’-CATTAATGCAGGTTGCGGCCGCATAGAGACACATCTCTTTCCG-3’
YPR196W-2-R	5’-CGGGCATTTAAATGCGGCCGCCCACTCTATTTATGTTCCGGCAAC-3’
YPR196W-2-RT-F	5’-CCTTCCCTTCGGTGAACAAC-3’
YPR196W-2-RT-R	5’-TGGTAGTGGTGGCGCTATTG-3’
YPR196W-2-pro-F	5’-CGCGTCGACGGTACCGAATTCACTTGATCTGAATATTTTCCTCTCC-3’
YPR196W-2-pro-F	5’-AACAGCCAAGCTCCGGATCCAGTCATTTGTAAAGTAAAATTCCAATAGA-3’

### Plasmids

The YCpG-TDH3pt vector was constructed as follows. The *TDH3* promoter and terminator were amplified by using primer pairs TDH3-pro-F + TDH3-pro-R and TDH3-term-F + TDH3-term-R, respectively. This construct was designed to introduce a unique *Stu*I site between the promoter and terminator. pJHXSB [20] was digested with *Asc*I, and then these three fragments (*THD3* promoter, *TDH3* terminator, and linearized pJHXSB fragments) were combined using the GeneArt Seamless Cloning and Assembly Enzyme Mix. The resulting plasmid was digested with *Asc*I, and the fragment containing the *TDH3* promoter, terminator and CEN/ARS was ligated with an *Asc*I fragment containing the G418-resistance gene from pYC030 [19] to yield the plasmid YCpG-TDHpt.

To construct the YCp-TEFpro-term vector, the *TEF2* promoter and terminator were amplified by using the primer pairs TEF2-pro-F + TEF2-pro-R and TEF2-term-F + TEF2-term-R, respectively. The YCpG-TDH3pt vector was digested with *Not*I, and then these three fragments were combined by using the GeneArt Seamless Cloning and Assembly Enzyme Mix to generate YCpG-TEF2pt. To construct the TEF2-Cas9 plasmid, Cas9 was amplified by using the primer pair Cas9-F plus Cas9-R with the CAS900A-1 plasmid (System Biosciences) as the template. The YCpG-TEF2pt vector was digested with *Stu*I, and this linearized vector then was combined with the amplified Cas9 gene by using the GeneArt Seamless Cloning and Assembly Enzyme Mix.

To construct YCpG-YRP196W-2, the *YRP196W-2* gene was amplified by PCR with the primer pair YRP196W-2-F + YRP196W-2-R using K1801 genomic DNA of as the template. YCpG-TDH3pt was digested with *Not*I, and then the linearized plasmid and the amplified *YRP196W-2* gene were combined by using the GeneArt Seamless Cloning and Assembly Enzyme Mix. To construct YCpG-YRP196W-2L, the *YRP196W-2L* gene was amplified by PCR with the primer pair YRP196W-2-F plus YRP196W-2-R using K1801M genomic DNA as the template. YCpG-TDH3pt was digested with *Not*I, and the linearized plasmid and the amplified *YRP196W-2L* gene were combined using the GeneArt Seamless Cloning and Assembly Enzyme Mix. The identity of the *YPR196W-2L* clone was confirmed by sequencing. To construct TDH3p-YPR196W-2, the *YPR196W-2* ORF was amplified by PCR with the primer pair TDH3-YRP196W-2-F plus TDH3-YRP196W-2-R using K1801 genomic DNA as the template. YCpG-TDH3pt was digested with *Stu*I, and the linearized plasmid and the amplified *YRP196W-2* gene were combined using GeneArt Seamless Cloning and Assembly Enzyme Mix, placing the ORF under control of the *TDH3* promoter.

To construct *MAL11*p*-lacZ*, pYC-Z130 [19] was digested with *Eco*RI and *Hin*dIII, and the *MAL11* promoter was amplified by PCR with the primer pair pYC-Z130-F + pYC-Z130-R using X2180-1A genomic DNA as the template. The linearized plasmid and the amplified promoter were combined using the GeneArt Seamless Cloning and Assembly Enzyme Mix.

To construct *YPR196W-2*p*-lacZ*, pYC-Z130 was digested with *Eco*RI and *Hin*dIII, and the *YPR196W-2* promoter was amplified by PCR with the primer pair YPR196W-2-pro-F + YPR196W-2-pro-R using K1801 genomic DNA as the template. The linearized plasmid and the amplified promoter were combined using the GeneArt Seamless Cloning and Assembly Enzyme Mix. The G418 resistance-encoding marker was then replaced with the *hygB* selection marker from plasmid pYC240 [19] by cloning via *Asc*I restriction and ligation.

### Phylogenetic analysis

An unrooted tree was constructed using Seaview version 4.4.2 software based on the ClustalW multiple alignment of DNA sequences, using neighbor-joining method with 1000 bootstrap counts [21, 22]. The accession numbers of the genes were as follows: *S*. *cerevisiae MAL23*, AF002704.1; *S*. *cerevisiae MAL43*, M81157.1; *S*. *cerevisiae MAL63*, AF003003.1; *S*. *cerevisiae MAL11*, CP020129.1; *S*. *cerevisiae MAL33*, CP020124.1; *S*. *cerevisiae YPR196W*, CP020138.1; *S*. *carlsbergensis MAL63*, M36537.1

### Ethyl methanesulfonate (EMS) treatment

Mutagenesis was performed using EMS as described in *Methods in Yeast Genetics* (Cold Spring Harbor Laboratory Course manual [23]. Briefly, exponentially growing K1801 was collected and then suspended in 5% EMS in Na-potassium buffer (pH 5.5). After 10 min, cells were collected, washed once with sterile water, and plated on an MA plate, which was incubated at 30°C. After 2–3 days, colonies were picked. The survival rate during mutagenesis was in the range of 50–55%.

### Reporter assay

Cells carrying a *lacZ* reporter plasmid were cultured in YPD medium containing 200 μg/mL G418. After the culture achieved exponential growth, cells were harvested and resuspended in YPD or YPM medium containing 200 μg/mL G418 and culturing was continued under the same conditions, with sampling at defined time points. β-galactosidase activity was measured using the yeast β-Galactosidase Assay Kit (Thermo Fisher Scientific).

### Test fermentation in SMal medium

Cells were cultured overnight in SD medium, and then cells were pelleted and resuspended in SMal medium at 1 × 10^7^ cells/mL and fermented at 20°C. Sugar content was measured using a refractometer and expressed in Brix unit at the indicated time point.

### Real-time polymerase chain reaction (RT-PCR)

Total RNA was extracted as described by Schmitt et al. [24]. cDNA synthesis was performed using Primescript Reverse Transcriptase (Takara), RT-PCR was performed using GoTaq qPCR Master Mix (Promega) on StepOnePlus Real-Time PCR system (Applied Biosystems).The RT-PCR primers used in this study are shown in Table 1.

### Small-scale *sake* test fermentation

Small-scale *sake* brewing tests were performed with a *sake* mash consisting of 72.8 g of α-preprocessed rice (Tokushima Seikiku, Tokushima, Japan) derived from a single-step pre-fermentation consisting of 80 g of white rice with a polishing rate at 70% (retaining the inner 70% of the grain), 19.2 g of dried rice *koji* (a culture of *A*. *oryzae* on steamed rice) (Tokushima Seikiku) derived from 20 g of white rice with polishing rate at 70%, 0.1 mL lactic acid (Wako), and 174 mL of water [25]. Each yeast strain was subjected to a two-step pre-cultivation consisting of incubation (at 30°C with rotary shaking for both steps), first in 3 mL of YPD medium for one day and then in 250 mL of YPD medium for a second day. The resulting yeast mycelia were washed twice with sterile water and inoculated into the mash to a density of 1.0 × 10^7^ cells per g of mash; the mixture was further incubated at 15°C for 18 days (from Day 1 to Day 19) without shaking, as described previously [25]. Progression of fermentation was monitored by measuring the culture’s weight at the same time each day; weight loss reflects CO_2_ emission from mash [26]. For serial sampling of mash at separate time points, multiple mash cultures with the same content were prepared and wholly collected at Day 5, 8, 11, 15, or 19. For sampling at earlier time points, multiple 1/10-scale mash samples were prepared and wholly collected at 0, 12, 24, or 48 h. At the designated time point, each sample was centrifuged at 5000 rpm (4620 g) at 15°C for 15 min. The resulting supernatants were subjected to the analyses described below. For sugar analyses, trichloroacetic acid (TCA) was added to each supernatant, immediately upon decanting, to yield a final concentration of 20% TCA in order to inhibit amylase activity.

### *Sake* metabolic profile

The concentrations of ethanol (and intermediates) during *sake* fermentation were measured by gas chromatography using a model GC 6890N machine (Agilent Technologies, CA, USA) equipped with a flame ionization detector (FID) and Agilent J&W DB-624 column (45 m × 0.543 mm; film thickness, 3 μm) [25]. Specific gravity (15/4°C), which reflects the sum of the alcohol and extractive content of *sake*, was measured with a density/specific gravity meter (DA-500; KEM, Kyoto, Japan); readings then were converted into *sake* meter values using the formula: *Sake* meter value = 1443/S-1443, where S = Specific gravity (15/4°C). In general, larger values on this scale indicate that fermentation has progressed further.

Acidity was determined by neutralization titration of 10 mL of *sake* with 0.1 N NaOH. Amino acidity was measured by the formol titration method [27]. The organic acid content was determined by high-performance liquid chromatography (HPLC) using a model LC-10AD (Shimadzu) machine equipped with a conductivity detector (Model CDD-10A; Shimadzu) and a Shim-pack SPR-H column (250 mm × 7.8 mm; Shimadzu). Aliquots (10 μL each) of samples were applied to the column using an automatic sampler SIL-10AD (Shimadzu) and eluted with 4 mM *p*-toluenesulfonic acid at a flow rate of 0.7 mL/min. The column oven temperature was 40°C. Volatile aromatic compounds were measured by headspace gas chromatography [28] using an Agilent 7694 Headspace Sampler and the GC 6890N (Agilent). Internal standards (consisting of *n*-amyl alcohol and methyl caprate) were added to aliquots of the samples prior to heating at 50°C for 30 min. Esters and higher alcohols then were separated using a DB-WAX capillary column (0.32 mm i.d. × 30 m, film thickness 0.25 μm; Agilent) after auto-injection of a headspace volume of 1 mL. The following conditions were applied: injection temperature, 200°C; oven temperature, 85°C; detector temperature, 250°C; and carrier gas (He), 2.2 mL/min.

Sugar content in the supernatants was determined by post-column labeling using phenylhydrazine with HPLC [29]. HPLC was performed with a Hitachi HPLC LaChrom Elite sugar analysis system (Hitachi High Technologies, Inc.), equipped with an auto sampler (L-2200), column oven (L-2300), pump (L-2130), FL detector (L-2485) and a column series consisting of a Shodex Asahipak NH2P-50 4E.

## Results

### The increased maltose fermentation ability of K1801M results from a one-base insertion in *YPR196W-2*

K7 is a well-known *sake* yeast, and the K7 genome sequence is available on a public website (http://www.bio.nite.go.jp/dogan/project/view/SC1) [16]. Notably, there are several K7-group strains that share a similar genetic background [30]. Analysis by our laboratory revealed that one of these strains, K1801, exhibits weak but measurable maltose fermentation ability, in contrast to the lack of maltose fermentation detected in other K7-group strains (S1 Fig). K1801 was derived as follows: first, K1601 was obtained from a cross between a K1001 segregant and a K7 segregant. Next, K1801 was obtained from a cross between a K1601 segregant and a K9 segregant (S2 Fig) [30]. To investigate the molecular basis of maltose fermentation by *sake* yeast in the present study, we chose to further characterize strain K1801, based on the fact that this strain shared the K7 genetic background but exhibited a detectable (albeit weak) background level of maltose fermentation (S1, S2 Figs).

**Fig 1:**
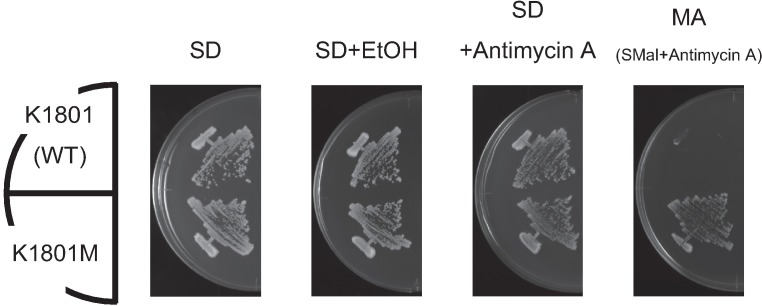
K1801M showed improved growth on maltose medium. Growth of K1801 and K1801M on various maltose-containing media. Each strain was streaked on SD, SD+EtOH (vehicle control), SD+Antimycin A, and MA (SMal+antimycin A) plates. Each plate was incubated at 30°C for 2–3 days.

To generate mutants showing high maltose fermentation, K1801 was treated with ethane methane sulfonate (EMS) and spread on MA plates (synthetic maltose medium containing 2 mg/L antimycin A). Antimycin A is known to inhibit the mitochondrial electron transfer system, permitting more precise assessment of the maltose fermentation ability of the resulting isolates [12]. Several mutants showing high maltose fermentation ability were obtained using this screening. One such mutant exhibited significant growth on the MA plate, in contrast to the parental K1801 (Fig 1, S3 Fig). This isolate, which we have designated K1801M, is the subject of further investigation in this study. When growth on SMal solid medium was monitored in parallel to that on MA solid medium (S3 Fig), K1801M exhibited obvious growth on both SMal and MA plates, whereas K1801 exhibited weak growth on the SMal plate and little growth on the MA plate, even at 6 days. Considering that maltose is taken up via a maltose-one-proton symport mechanism [31], and that ATP hydrolysis by the plasma membrane ATPase is required to expel the protons that enter the cell together with maltose [32, 33], the observed growth difference suggested that K1801 can respire maltose weakly, but an extra ATP, derived from the mitochondrial electron transfer system, is required for efficient maltose transport. To exclude the mitochondrial effect in this study, MA plates were used to evaluate maltose fermentation ability in subsequent experiments.

**Fig 2:**
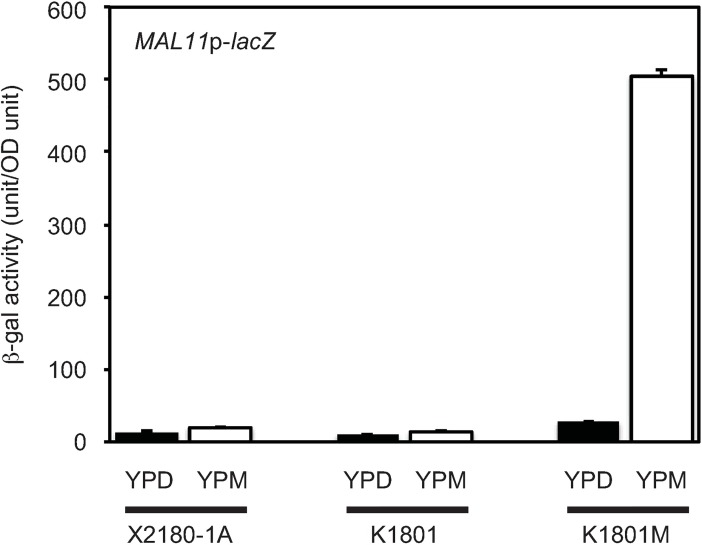
K1801M showed enhanced expression of *MAL11*p*-lacZ* reporter gene. Cells carrying *MAL11*p*-lacZ* reporter plasmid were harvested after overnight cultivation in YPD medium, then transferred to YPD or YPM medium, and further cultured at 30°C for 2 h. β-galactosidase activity was measured using yeast β-galactosidase assay kit. X2180-1A, which does not have a functional MALR [5], was used as a negative control.

In the K7 genome sequence database, the genes *MAL11*, *MAL13*, *MAL31*, *MAL32*, *MAL33*, *YPR196W* and *YPR196W-2* are annotated as members of the *MAL* gene family. Comparison of these gene sequences to those of the respective S288C homologs revealed that the K7 *MAL33* gene, which encodes a MALR, harbors a single nucleotide deletion (S4 Fig); no such deletion was observed in the paralogous K7 *MAL13* and *YPR196W* genes. Therefore, it is unlikely that *MAL13* or *YPR196W* confer maltose fermentation ability to K1801. In addition, *MAL33* gene sequence in K1801 harbors the nucleotide deletion identical to that in K7. These data collectively suggest that *MAL33*, but not *MAL13* nor *YPR196W*, is functionally associated with the increased maltose fermentation activity in K1801M strain (Fig 1, S3 Fig). In order to compare MALR activity in K1801 with that in K1801M, we introduced into both strains a *MAL11*p*-lacZ* reporter plasmid, in which the *MAL11* promoter was fused with the *lacZ* gene. This construct permitted the monitoring of MALR activities, given that expression of *MAL11-lacZ* depends on *trans*-activation by MALR proteins [34]. Notably, while *MAL11*p*-lacZ* reporter activity was repressed in medium containing glucose, expression of this reporter was induced (in K1801M only) in medium containing maltose (Fig 2). These results indicated that K1801M has a maltose-inducible MALR for *MAL11* compared to K1801. However, a candidate *MALR*, *MAL33*, in K1801M strain had the same mutation (relative to S288C) as that in the *MAL33* gene from K7 and K1801 strains, suggesting that a MALR other than the *MAL33* protein mediates the elevated maltose fermentation ability observed in K1801M.

To clarify the increased *trans*-activation of *MAL11* observed in K1801M, we explored genetic alterations in other MALR-encoding genes in K1801M relative to the K1801 parent. As noted below, we ultimately focused on *YPR196W-2*. This gene is annotated in the K7 genome database, but not in the *Saccharomyces cerevisiae* Genome Database (SGD), and was designated *YPR196W-2* because the K7 gene shows highest sequence similarity to *YPR196W* (*MALR*) among genes in the S288C genome. The Ypr196W-2p sequence was analyzed by BLAST search, and found to be structurally similar to several MALR proteins (including Mal23p, Mal43p, and Mal63p). Gibson et al. [3] reported that MALR proteins like Mal23p, Mal43p, and Mal63p have high *trans*-activation ability. The *MALR* genes in the S288C strain and the K7 strain are summarized in Table 2.

**Table 2:** Chromosomally encoded *MALR* genes in each yeast.

S288C strain	*MAL13* (Chr7), *MAL33* (Chr2), *YPR196W* (Chr16)
K7 strain	*MAL13* (Chr7), *MAL33* (Chr7), *YPR196W* (Chr16), *YPR196W-2* (Chr7)

**Fig 3:**
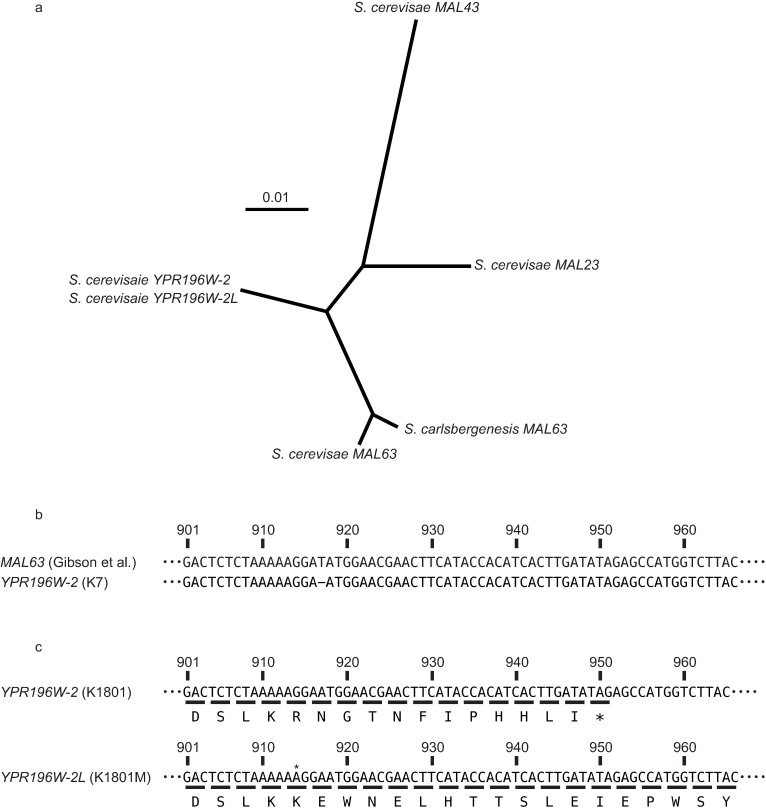
Phylogenetic and sequence analysis of *YRP196W-2* in K1801 and K1801M. **a** An unrooted phylogenetic tree of selected *MALR* genes was constructed using the Neighbor-Joining Method (1000 replicates) in the CLUSTALW multiple alignment program [21, 22]. Bar = 0.01 nucleotide substitutions per site. **b** Sequence comparison of *MAL63* [3] and *YRP196W-2*. **c** DNA (upper line) and protein (bottom line) sequences of *YRP196W-2* and *YRP196W-2L*. The asterisk indicates the nucleotide insertion detected in K1801M.

*MAL23*, *MAL43*, and *MAL63* each encode a polypeptide consisting of 470 amino acids; in contrast, the *YPR196W-2* ORF is only 316 amino acids long. *YPR196W-2* was the closest to *MAL63* (Fig 3A) and multiple alignments of *YPR196W-2* and *MAL63* revealed that the K7 *YPR196W-2* has (compared to *MAL63*) a single nucleotide deletion at a position corresponding to nucleotide position 917 of the *MAL63* ORF (Fig 3B). This deletion results in a frameshift compared to *MAL63*, such that the *YPR196W-2* encodes a MALR protein that is C-terminally truncated relative to Mal63p. The sequence of *YPR196W-2* in K1801 was identical to that in the K7 strain. However, some of the *YPR196W-2* sequences amplified from K1801M harbored a single nucleotide insertion in the ORF compared to the K1801 sequence. Specifically, for three out of ten clones obtained by PCR from K1801, an adenine insertion was observed at nucleotide 914 of the *YPR196W-2* ORF, which therefore encoded a mutant Ypr196W-2p protein 470 amino acids long, a size that is comparable to those of Mal23p, Mal43p, and Mal63p (Fig 3C). The remaining seven clones possessed *YPR196W-2* sequences identical to that of K1801. The observed ratio of mutant alleles (3/10) was consistent with the previous proposal that the K1801 strain is a triploid [35]. Indeed, we were able to confirm, using flow cytometry, that both K1801 and K1801M are triploids. These results indicated that a single nucleotide insertion in *YPR196W-2* occurred on one of three copies of Chromosome 7 in K1801M. Hereafter, we refer to the mutated allele of *YPR196W-2* in K1801M as *YPR196W-2L* (Long). We also detected other single nucleotide insertions in proximity to ORF nucleotide position 917 in other maltose-assimilative mutants (referred to as K1801M’ and K1801M” in S5 Fig). These results support the notion that extension of the *YPR196W-2L* ORF by a single nucleotide insertion is associated with enhanced maltose fermentation.

### Different functionalities of *YRP196W-2* and *YRP196W-2L*

**Fig 4:**
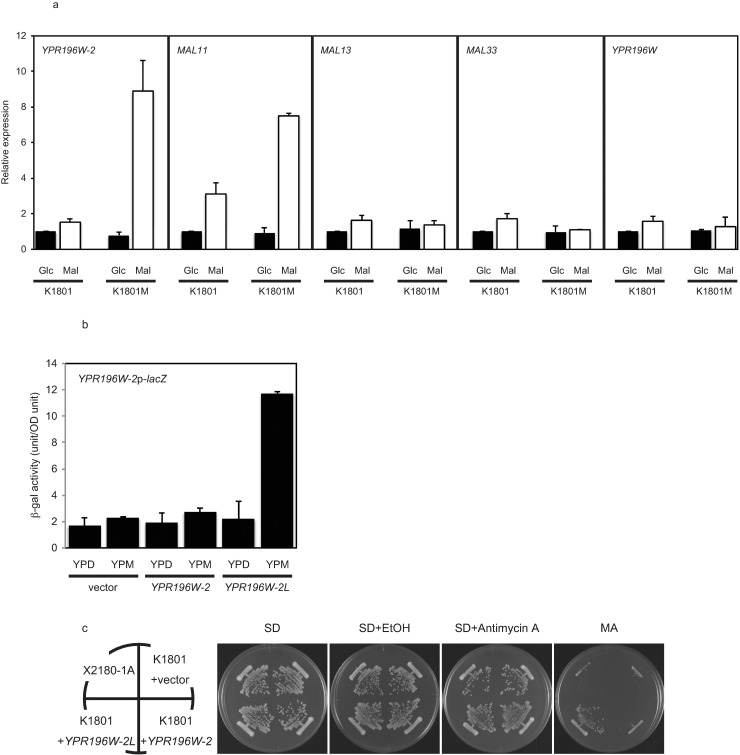
Expression of *YRP196W-2* /*-2L* and *MAL11* target gene in K1801M. **a** Gene expression in K1801 and K1801M. Cells were harvested after overnight cultivation in YPD medium, then transferred to YPD (indicated as Glc) or YPM (indicated as Mal) and cultured at 30°C. After 6 h, total RNA was extracted and RT-PCR was performed using the indicated primers. The mRNA transcript level of each target gene was standardized by normalizing the value to that obtained for the control gene *TDH3*, and then presented as the value relative to that observed for K1801 grown on YPD. Data are presented as mean and standard deviation from three independent experiments. **b** Cells harboring a *YPR196W-2*p*-lacZ* reporter in a K1801 background were harvested after overnight cultivation in YPD medium, then transferred to YPD or YPM and cultured at 30°C for 6 h. β-galactosidase activity was measured using yeast β-galactosidase assay kit. **c** Restoration of maltose fermentation ability by *YPR196W-2L*. Cells carrying the indicated plasmid were streaked on SD, SD+EtOH (vehicle control), SD+Antimycin A, and MA (SMal+antimycin A) plates. Each plate was incubated at 30°C for 2–3 days.

The gene expression of *YPR196W-2* and *YPR196W-2L* was analyzed by real-time polymerase chain reaction (RT-PCR) using total RNA extracted from cells cultured for 6 h in YPD (containing glucose as the sole carbon source) or YPM (containing maltose as the sole carbon source) medium. The level of *YPR196W-2* mRNA in K1801 did not differ between YPD and YPM, whereas the level of *YPR196W-2L* mRNA in K1801M was elevated in YPM compared to that in YPD (Fig 4A). The expression of other MALRs, *MAL13*, *MAL33*, *YPR196W*, in K1801M were same as K1801 (Fig 4A).

Since the expression of a major maltose transporter-encoding gene, *MAL11*, is known to be induced in a MALR-dependent manner [13], we used *MAL11* as a reporter gene for MALR activity. Notably, *MAL11* expression was significantly higher in K1801M than in K1801 (Figs 2 and 4A). This result indicated that *YPR196W-2L* induces the expression of *MAL11*, whose promoter has MAL-activator binding sites [13]. Furthermore, *YPR196W-2* itself was also induced by maltose (Fig 4A), a result consistent with the observation that the *YPR196W-2* promoter also contains MAL-activator binding sites (S6 Fig; MGCNNNNNNNNNMGS, where M = adenine or cytosine, S = guanine or cytosine, and N = any of the four deoxyribonucleotides [36, 37]). In the *YPR196W-2* promoter, two overlapping MAL-activator binding sites were observed at nucleotide positions (relative to the translation start) -178 to -164 and -174 to -160 (S6 Fig, where the two sites are designated MAL BD1 and MAL BD2, respectively). The presence of these MAL-activator binding sites in the *YPR196W-2* promoter suggests that *YPR196W-2* is itself positively regulated by a MALR. Since the promoter of *YPR196W-2*/-*2L* was found to contain putative MAL-binding *cis*-elements, *trans*-activation of *YPR196W-2/-2L* expression by the encoded protein was tested by *lacZ* reporter assay in K1801. A chimeric reporter gene, in which the *lacZ* gene was fused with the native promoter of *YPR196W-2/-2L* (representing 600 bp of sequence extending upstream from the translation start site), was introduced into the yeast along with a plasmid expressing *YPR196W-2* or *YPR196W-2L* under the regulation of the native promoter. In YPD medium, reporter activity in yeast harboring *YPR196W-2* or *YPR196W-2L* was statistically indistinguishable from that obtained in a strain harboring the reporter construct along with empty vector (Fig 4B). Notably, reporter activity was significantly increased in maltose-grown yeast harboring the reporter in combination with *YPR196W-2L*, but not in maltose-grown yeast harboring the reporter in combination with *YPR196W-2*.

To exclude the possibility that the maltose fermentation activity of K1801M reflects mutations in genes other than *YPR196W-2*, *YPR196W-2* and *YPR196W-2L* were separately introduced into the parent K1801 strain, and these transformants were evaluated for growth on MA plates. As expected, the yeast expressing *YPR196W-2L* showed enhanced maltose fermentation ability, unlike yeast expressing *YPR196W-2* (Fig 4C). The improved growth of yeast expressing *YPR196W-2L* on MA plate mirrored the previous observation that *MAL11* was induced to higher levels in K1801M than in K1801 (Fig 2). Together, these results indicated that the basal level of *MAL11* expression in K1801 is not sufficient to support growth on the MA plate; in contrast, the mutant *YPR196W-2L* gene is sufficient for growth on this medium.

These results showed that (1) Ypr196W-2Lp is able to exert autoregulatory *trans*-activation at the *YPR196W-2L* promoter, (2) the C-terminal domain of this MALR is required for *trans*-activation of *MAL11*, and (3) the gene expression of *YPR196W-2L* is responsive to maltose.

### *YPR196W-2* alleles are associated with maltose fermentation ability

**Fig 5:**
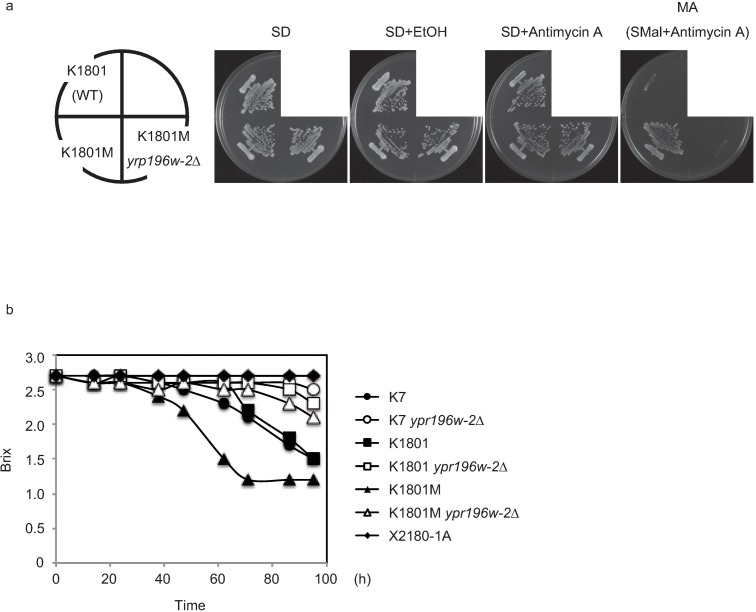
The maltose fermentation ability of *ypr196W-2*Δ *sake* yeast. **a** Growth of K1801 and its derivatives on maltose-containing media. Each strain was streaked on SD, SD+EtOH (vehicle control), SD+Antimycin A, and MA (SMal+antimycin A) plates. Each plate was incubated at 30°C for 2–3 days. **b** Maltose fermentation test of *ypr196W-2*Δ strain. Cells were harvested after overnight cultivation in SD medium, then resuspended in SMal medium at 1 × 10^7^ cells/mL and cultured at 20°C. Sugar content in the medium was measured at the indicated times.

As shown in Fig 4C, the introduction of *YPR196W-2L* was sufficient to confer maltose fermentation ability in the *sake* yeast K1801. To clarify the contribution to maltose fermentation ability of the endogenous *YPR196W-2* alleles, the wild-type genes were deleted in K1801M. As it was technically difficult to selectively disrupt only the genes corresponding to the *YPR196W-2* allele (given that *YPR196W-2* and *YPR196W-2L* differ by a single nucleotide polymorphism), these allelic *MALR* genes were disrupted simultaneously using a CRISPR/Cas9 gene editing system in the triploid K1801M strain. Like K1801, the resulting K1801M *ypr196W-2*Δ strain (null) showed no maltose fermentation ability on an MA plate (Fig 5A). This result demonstrated that a single nucleotide insertion at *YPR196W-2* permits *YPR196W-2L* to function as a dominant positive regulator for maltose fermentation in K1801M.

Gibson et al. [3] previously demonstrated that a C-terminally truncated Mal63p, 283 amino acids in length, still possessed the ability to induce the maltase-encoding gene, although the level of induction with this shortened protein was lower than that observed with the full length (470-amino acid) Mal63p, indicating that a C-terminally truncated MALR may maintain partial function. Similarly, wild *sake* yeasts are known to exhibit weak maltose fermentation activity (S1 Fig), suggesting that the C-terminally truncated (316-amino acid) Ypr196W-2p also retains partial function in *sake* yeast. To test whether the weak maltose fermentation observed in *sake* yeast depends on the truncated MALR Ypr196W-2p, the sugar consumption rate of yeast cells under maltose fermentation conditions was determined by measuring sugar content using a refractometer. In control experiments, K7 and K1801 strains showed slow maltose consumption (Fig 5B). In contrast, the maltose consumption rate of K1801M was faster than those of the control strains. When *YPR196W-2* was disrupted in K1801 or K1801M, the resulting mutants consumed maltose at rates lower than those of the control strains. Similar data were obtained in the K7 genetic background (Fig 5B). These results showed that *YPR196W-2* is responsible for the weak maltose fermentation ability of this *sake* yeast under maltose fermentation conditions. As shown in Fig 5, the K1801M *ypr196w-2*Δ strain failed to grow on an MA plate, but was viable in maltose-supplemented fermentation conditions, a difference that presumably reflects the decreased availability of the mitochondrial electron transfer system in the presence of antimycin A. This result also is consistent with the case in strain K1801 (Fig 1, S3 Fig). These results indicated that K1801M *ypr196w-2*Δ still had a weak maltose fermentation ability that is dependent upon the mitochondrial electron transfer system, suggesting that another functional *MALR* (other than *YPR196W-2*) that is present in K1801 provides this very weak MALR activity.

**Fig 6:**
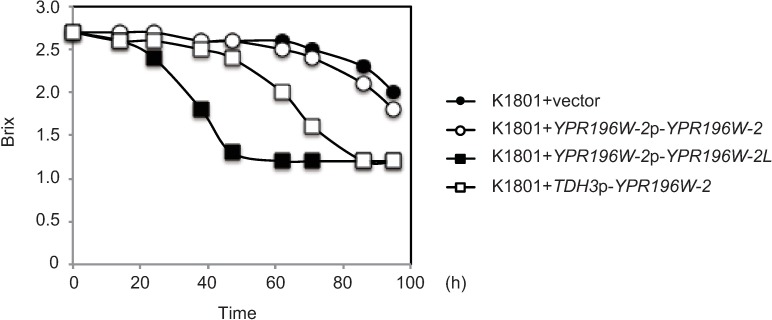
Maltose fermentation ability of cells carrying *TDH3p*-driven *YRP196W-2*. Maltose fermentation test of cells carrying *TDH3*p-*YPR196W-2*. Cells carrying each plasmid were harvested after overnight cultivation in SD medium, then transferred into SMal medium at 1 × 10^7^ cells/mL and cultured at 20°C. Sugar content in the medium was measured at the indicated times.

To assess the relationship between the levels of the MALR proteins and maltose fermentation, maltose consumption rates were determined in yeasts expressing distinct alleles of this *MALR* gene under the control of promoters of various strengths. The maltose consumption rate of the cells harboring a construct (*TDH3*p-*YPR196W-2*) driving constitutive high-level expression of the truncated protein was lower than that obtained with *YPR196W-2L* under control of the endogenous *YPR196W-2* promoter (*YPR196W-2*p-*YPR196W-2L*), but the *TDH3p*-*YPR196W-2* maltose consumption rate was still higher than that obtained with *YPR196W-2* under control of the endogenous *YPR196W-2* promoter (*YPR196W-2*p-*YPR196W-2*) (Fig 6). Taken together, these results indicated the following: (1) exogenous expression of *YPR196W-2L* is sufficient to confer maltose fermentation ability to K1801 in SMal medium, and (2) the restored C-terminal domain in Ypr196W-2Lp is crucial for full function as a MALR, such that overexpression of the truncated protein is not sufficient to compensate for the attenuated activity of Ypr196W-2p.

**Fig 7:**
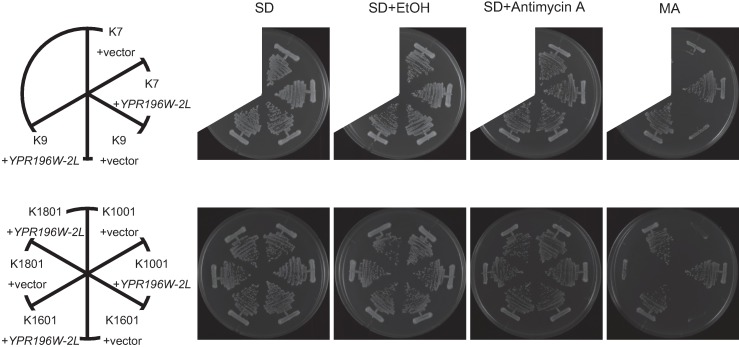
Growth in the presence of maltose of K7 family strains carrying *YRP196W-2L*. Each strain was streaked on SD, SD+EtOH (vehicle control), SD+Antimycin A, and MA (SMal+antimycin A) plates, and plates were incubated at 30°C for 2–3 days.

K7 *sake* yeast family members are presumed to share the preponderance of their genetic background with K1801; however, maltose fermentation ability was found to differ among various members of the K7 *sake* yeast family (S1 Fig). K7-family *sake* yeast, which share the *YPR196W-2* allele shown in Fig 3B, failed to grow on an MA plate, presumably due to the lack of maltose-inducible MALRs. Notably, exogenous introduction of *YPR196W-2L* into various K7-family strains was sufficient to confer maltose fermentation ability to these *sake* yeast strains (Fig 7).

**Fig 8:**
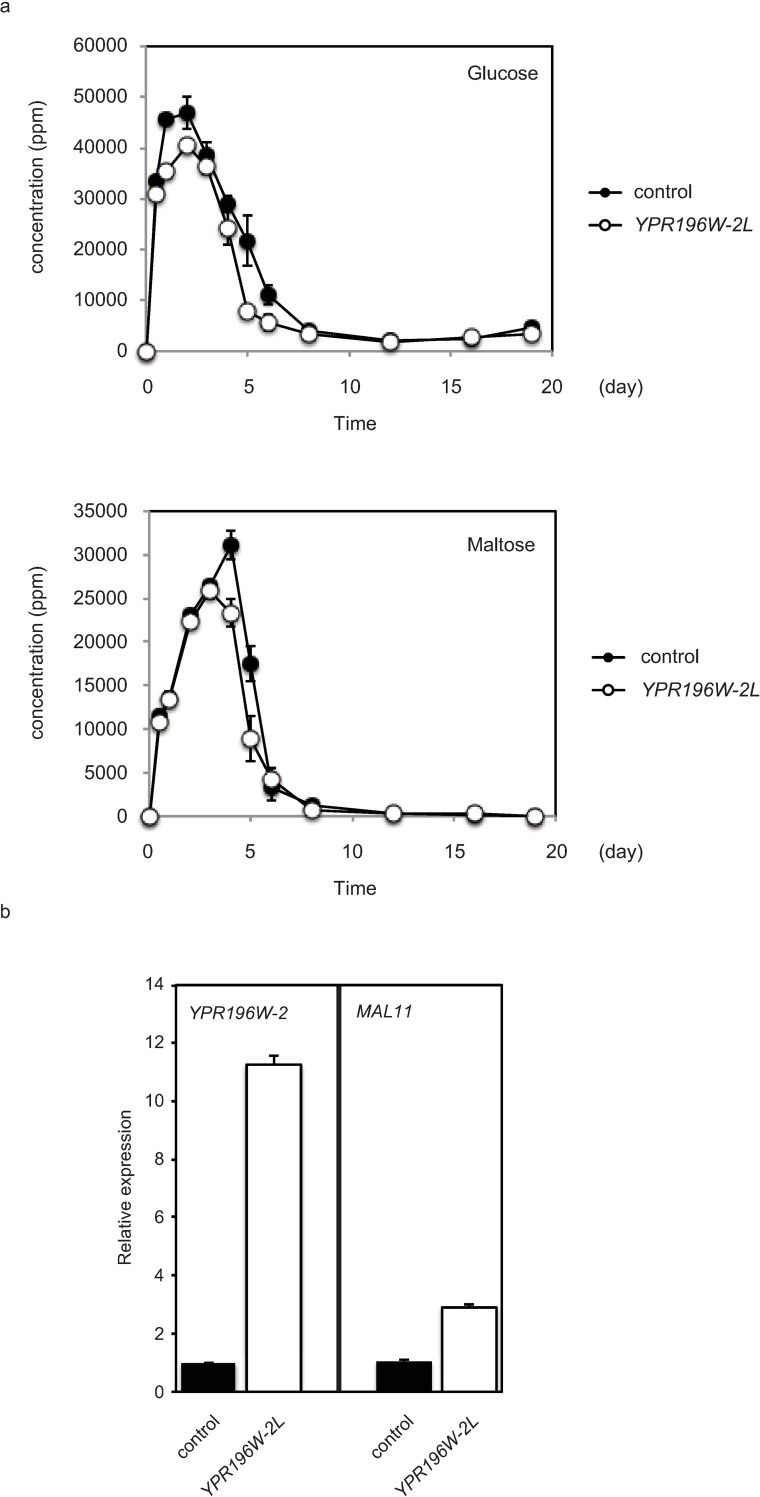
Cells expressing *YPR196W-2L* exhibit rapid maltose incorporation compared to control in a small-scale *sake* test fermentation. **a** *Sake* mash that contained yeast cells with control vector or *YPR196W-2L* were prepared triplicate as described in the Materials and Methods section. Glucose and maltose content in the test fermentation. Mash was sampled at the indicated time point, and the supernatant was collected by centrifugation and assessed for sugar content. **b** At Day 3, total RNA was extracted and RT-PCR was performed using the indicated primers. The mRNA transcript level of each target gene was standardized by normalizing the value to that obtained for the control gene *TDH3*, and then presented as the value relative to that observed for control (empty vector) strain. Data are presented as mean and standard deviation from three independent experiments.

*Sake* fermentation is notable for the incorporation of parallel catalytic pathways, including simultaneous *Aspergillus*-mediated saccharification and yeast-mediated fermentation. To assess the practical impact on *sake* fermentation of *YPR196W-2L*, small scale *sake* fermentation (total rice amount: 100g) was performed with *sake* yeast (K1801 strain) transformed with *YPR196W-2L* or with control empty vector. Based on CO_2_ emission, yeast expressing *YPR196W-2L* showed slightly faster fermentation than that harboring control vector (S7 Fig). A large amount of maltose was generated during Days 2–4 of fermentation, presumably due to saccharification of rice starch (Fig 8A lower panel). The amount of maltose in the *sake* mash was lower in the *YPR196W-2L*-expressing yeast than in the control yeast, which was consistent with the enhancement of maltose fermentation activity by the *YPR196W-2L* allele. Moreover, the amount of glucose also was lower with the *YPR196W-2L*-expressing yeast (compared to the control strain). This result presumably reflects the decreased availability of maltose (for degradation into glucose by *A*. *oryzae*) in the *sake* mash (Fig 8A upper panel). Concomitantly, *MAL11* was highly expressed at Day 3 in the *YPR196W-2L*-expressing strain than in the control yeast (Fig 8B). However, final alcohol production by yeast expressing *YPR196W-2L* was similar to that of the control (S1 Table). It should be noted that maltose consumption temporally followed glucose consumption, indicating that the glucose repression system was active during *sake* fermentation. Chemical analysis of organic compounds produced during fermentation showed that there were no remarkable changes in the levels of those compounds when comparing between the strains harboring *YPR196W-2L* or empty vector (S1 Table). Therefore, *sake* yeast carrying an ectopic copy of *YPR196W-2L* is expected to exhibit moderately accelerated fermentation without apparent changes in *sake* quality. These data showed that *YPR196W-2L* plays a potential role in maltose fermentation in general.

## Discussion

**Fig 9:**
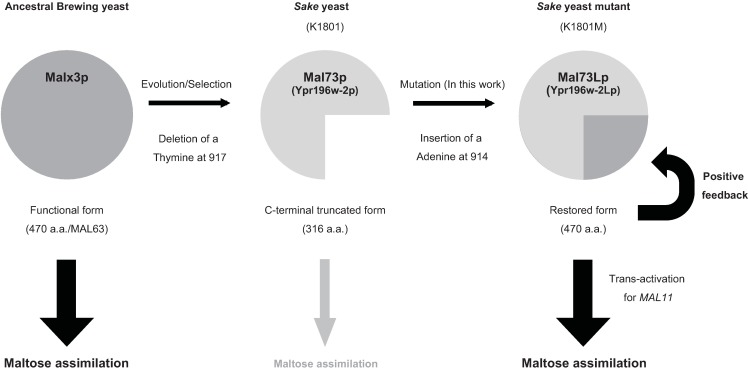
Summary of this work. An ancestral brewing yeast is presumed to have harbored a functional Mal63p ortholog. During the evolution or human selection of this ancestral strain, *MAL63* experienced the deletion of a residue at nucleotide position 917 of the ORF. The resulting gene, *MAL73*, encodes a truncated MALR protein with attenuated *trans*-activation activity. As shown in this study, K1801M, isolated from K1801 on the basis of enhanced maltose fermentation ability, harbors a *MAL73* gene in which a single nucleotide was inserted at ORF residue 914, resulting in a frameshifted extension of the ORF. The resulting *MAL73L* gene encodes a functionally restored MALR with improved *trans*-activation activity compared to the parental Mal73p.

In this study, we isolated a mutant *sake* yeast strain that showed enhanced maltose fermentation ability (Fig 9). A single nucleotide insertion in a *MALR* gene, *YPR196W-2*, was responsible for the elevated level of maltose fermentation; introduction of the mutant version of *YPR196W-2*, designated *YPR196W-2L*, was sufficient to confer enhanced maltose fermentation to the original *sake* yeast strain K1801. Conversely, deletion of *YPR196W-2* in K1801 lowered the strain’s maltose fermentation ability below that of K1801. *YPR196W-2* is structurally similar to *MAL63*, but (compared to other MALR proteins) lacks a thymine at nucleotide position 917 of the ORF. This single nucleotide deletion presumably occurred in the ancestral strain during differentiation and selection for *sake* fermentation; the resulting truncated form of Ypr196W-2p is apparently hypomorphic, resulting in decreased maltose fermentation ability. Together with the additional experiments (Figs 4–8), our data demonstrated that *YPR196W-2* is the major cause of the attenuated maltose fermentation ability of *sake* yeast. We propose redesignating *YPR196W-2* as *MAL73* to indicate its functional association with maltose fermentation.

Brown et al. [38] showed that *MAL* genes are located in subtelomeric regions and implied that different yeast strains harbor different alleles and copy numbers of the various *MAL* genes. *MALR* genes are classified into three clades, including a *MAL13*-like clade, a *YPR196W* clade, and a *MAL63*-like clade. *MAL73*, the gene characterized in this study, is classified into the *MAL63*-like clade based on phylogenetic analysis (Fig 3A). Moreover, the Verstrepen group also showed that a difference in DNA-binding domains between Malx3p and Yfl052Wp, notably that at amino acid position 12 (Arg in Malx3p, Cys in Yfl052Wp), was responsible for the distinct DNA-binding specificity of these MALRs [39]. Like Malx3p, Mal73p encodes an Arg residue at position 12, suggesting that *MAL73* is evolutionarily derived from a *MALx3*-type *MALR* with structural similarity to *MAL63*, and further indicating that the ancestral *sake* yeast exhibited a sugar preference for maltose.

### Mal73p lacks C-terminal activation domain due to a single nucleotide deletion

The MALR protein has a DNA binding domain at amino acid residues 1–100, a *trans*-activation domain at residues 60–283, and a maltose responsive domain at the C-terminus [4]. Gibson et al. [3], Hu et al. [4], and Danzi et al. [40] previously reported that mutants that are not inducible by maltose frequently are the result of missense mutations or short deletions within the C-terminal regulatory domain. Moreover, the C-terminal domain is presumed to take part in the formation and/or maintenance of an active state of MALR [41]. The insertion of a single nucleotide insertion in *MAL73* resulted in a frameshift that endowed Mal73Lp with an elongated C-terminal domain compared to Mal73p, such that Mal73Lp exhibited enhanced regulatory activity. Our results showed that *MAL11* is specifically induced in maltose-grown K1801M, which encodes the elongated Mal73Lp, but not in the maltose-grown parent K1801, which encodes a shorter (relative to Mal63p) protein. This observation is consistent with a previous report that a truncated Mal63p, consisting of 283 amino acids, shows weaker induction of a maltase-encoding gene than the full-length (470-amino acid) Mal63p [3]. Furthermore, *MAL73L* showed higher self-*trans*-activation via its own promoter than did *MAL73* (Fig 4B). In contrast, overexpression of *MAL73* by the *TDH3* promoter slightly improved maltose fermentation in K1801 (Fig 6), again demonstrating the functional significance of the C-terminal domain of MALR in *trans*-activation (Fig 9).

Among the tested *sak*e yeast strains, deletion of *MAL73* decreased efficiency of maltose consumption compared to the respective parent (Fig 5B), showing that the weak maltose fermentation ability of *sake* yeast depends on *MAL73*. However, these *sake* yeast strains did not completely lose maltose fermentation ability, retaining a basal level of maltose fermentation higher than that of the laboratory strain X2180-1A. Therefore, we infer that there exist additional (beyond Mal73p) MALRs and/or MALR activation mechanisms in K1801.

### Biological significance of *YPR196W-2* allele

The genome of the ancestral yeast strain is inferred to have harbored five paralogous *MAL* loci. During subsequent descent, those *MAL* loci presumably evolved in response to changes in natural environments and nutrient supplies, and under human selection for specialized fermentations with various plant materials. Some descendants lost entire loci from their genomes, as was the case of *MAL2/4/6* in maltose fermentable yeasts [10, 42]. In other strains, *MAL* genes were altered in expression or function, as was the case with *MAL13* or *MAL33* in S288C [5, 6]. In this study, we showed that *sake* yeast encodes a MALR (Mal73p) that is truncated and exhibits decreased regulatory function (compared to other known MALRs). Presumably, at some point in the past, an ancestral *MALR* gene suffered a single nucleotide deletion, resulting in a frameshift; this mutation introduced a premature stop codon, yielding a C-terminally truncated MALR protein. *Sake* fermentation characteristically employs continuous degradation of rice starch into glucose via the action of α-amylase and glucoamylase provided by the fungus *A*. *oryzae*. This process occurs in parallel with saccharification, whereby maltose is generated before immediately being further degraded into glucose monomer units (Fig 8A). Together, these processes permit alcoholic fermentation through catalysis by *sake* yeast. Thus, *sake* yeast are presumed to have adapted for the specialized situation wherein a large amount of glucose is provided as a carbon source. Liti et al. [43], Schacherer et al. [44] and Peter et al. [45] have described phylogenies of *Saccharomyces cerevisiae* in which *sake* yeasts cluster separately as a distinct clade. These results were presumed to reflect, in part, the unique evolution of *sake* yeast in Japanese archipelago as a consequence of adaptation and selection for *sake* fermentation. With respect to maltose fermentation, *MAL73* gene might be a beneficial trait to retain full ability of maltose fermentation by insertion of a single nucleotide when that ability is required.

Introducing the *MAL73L* allele into *sake* yeast facilitated slightly improved maltose fermentation in small *sake* fermentation as well as in SMal medium. Based on temporal CO_2_ emission and differential sugar consumption (Fig 8A, S6 Fig), maltose fermentation follows glucose fermentation at Days 2–4 of *sake* fermentation, suggesting that *MAL73L*-mediated maltose fermentation initiates earlier than Day 4, presumably due to release from glucose repression. This notion is supported by the observation that *MAL73L* is inactive in the presence of high glucose concentrations (Fig 4). The slight enhancement of maltose fermentation by *MAL73L* seems to be practically beneficial for faster *sake* fermentation; ectopic expression of this allele did not appear to impose obvious metabolic alterations in the characteristic organic compound profile that we evaluated. Our findings indicate that the *MAL73L* gene may serve as a valuable genetic tool for improvement of maltose fermentation via the insertion of a single nucleotide in a *MALR* gene.

Notably, all of the *sake* yeasts surveyed in this work encoded Mal73p as a C-terminally truncated hypomorphic protein (compared to other MALR proteins), suggesting a founder effect derived from an ancestral *sake* yeast and/or a trace of historical human selection for the hypomorphic allele. Domestication of *sake* yeasts may have accompanied by genetic traits involving a specific organic compound profile in the fermented products as well as stable fermentation, since such a profile would have significantly altered the flavor and quality of *sake* during traditional-to-industrialized *sake* history. An obvious beneficial effect of the hypomorphic allele on *sake* quality is currently unknown, however future large-scale *sake* fermentations and subsequent sensory and chemical evaluations would clarify this issue.

## Supporting information

S1 Fig Maltose assimilation ability of K7 series strains. Maltose assimilation ability of *sake* yeast under fermentation conditions. Cells were harvested after overnight cultivation in SD medium, transferred into SMal medium at 1 × 10^7^ cells/mL, and further cultured at 20°C. Sugar content in the medium was measured at the indicated times.
(TIF)

S2 Fig K1801 breeding tree. K1801 was bred as follows: first, K1601 was bred by the mating of a K1001 segregant with a K7 segregant. Next, K1801 was bred by the mating of a K1601 segregant and a K9 segregant.
(TIF)

S3 Fig K1801 and K1801M growth on each plate. Each strain was streaked on SD, SD+EtOH (vehicle control), SD+Antimycin A, SMal, SMal+EtOH (vehicle control) and MA (SMal+antimycin A) plates. Each plate was incubated at 30°C, and growth was monitored daily over the course of 2–6 days.
(TIF)

S4 Fig *MAL33* structure in S288C and K7. The *MAL33* (S288C) sequence was downloaded from the Saccharomyces genome database (http://www.yeastgenome.org/), and the *MAL33* (K7) sequence was downloaded from the DOGAN–*Saccharomyces cerevisiae* K7 database (http://www.bio.nite.go.jp/dogan/project/view/SC1)
(TIF)

S5 Fig The *YPR196W-2* sequence from other maltose-assimilative, K1801-derived strains. DNA (upper line) and amino acid (bottom line) sequences of *YPR196W-2* homologs from other maltose-assimilative K1801-derived strains (K1801M’ and K1801M”) that were isolated through the mutagenesis screen of K1801 described in the Materials and Methods section. An asterisk is used to indicate the inserted or changed nucleotide in each strain.
(TIF)

S6 Fig The promoter sequence of *YPR196W-2*. MAL-activator binding sites are indicated as MAL BD1 and MAL BD2. The consensus sequence of the MAL-activator binding site is MGCNNNNNNNNNMGS, where M is adenine or cytosine, S is guanine or cytosine, and N is any of the four deoxyribonucleotides.
(TIF)

S7 Fig Cells expressing *YPR196W-2L* exhibited accelarated fermentation compared to control in a small-scale *sake* test fermentations. *Sake* mash containing yeast cells with control vector or *YPR196W-2L* plasmid were prepared in triplicate as described in the Materials and Methods section. Weight loss of the mash (reflecting CO_2_ gas emission) was measured at the same time each day. The upper panel shows total cumulative CO_2_ gas emission, and the bottom panel shows CO_2_ gas emission per day. Data are presented as the mean and standard deviation from three independent experiments.
(TIF)

S1 Table Effects of expression of *MAL73L* gene on *sake* brewing characteristics (triplicated assay). (DOCX)
