# Supplementary figures and images for: MAL73, a novel regulator of maltose fermentation, is functionally impaired by single nucleotide polymorphism in sake brewing yeast
Source: PLoS One. 2018 Jun 12;13(6):e0198744. doi: 10.1371/journal.pone.0198744 (PMC5997316; doi:10.1371/journal.pone.0198744)

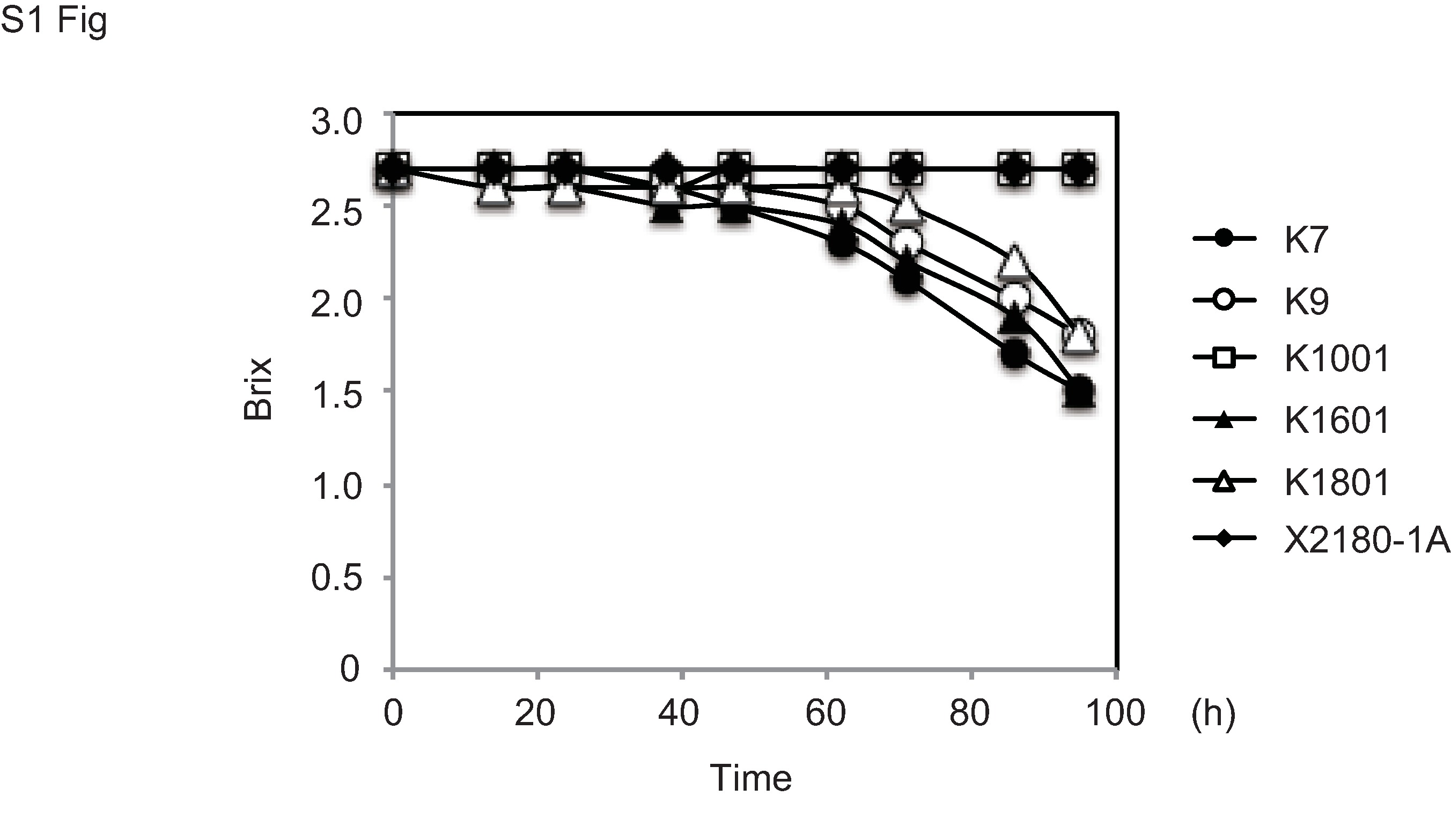

Supplement: S1 Fig — Maltose assimilation ability of sake yeast under fermentation conditions. Cells were harvested after overnight cultivation in SD medium, transferred into SMal medium at 1 × 10^7^ cells/mL, and further cultured at 20°C. Sugar content in the medium was measured at the indicated times. (TIF) [file pone.0198744.s001.tif]

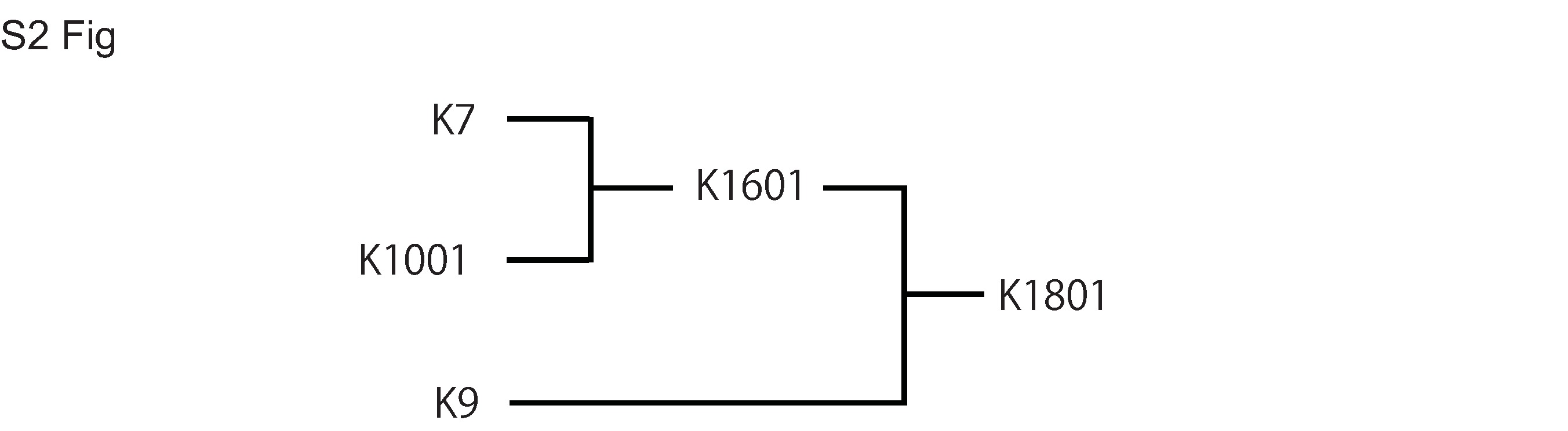

Supplement: S2 Fig — K1801 was bred as follows: first, K1601 was bred by the mating of a K1001 segregant with a K7 segregant. Next, K1801 was bred by the mating of a K1601 segregant and a K9 segregant. (TIF) [file pone.0198744.s002.tif]

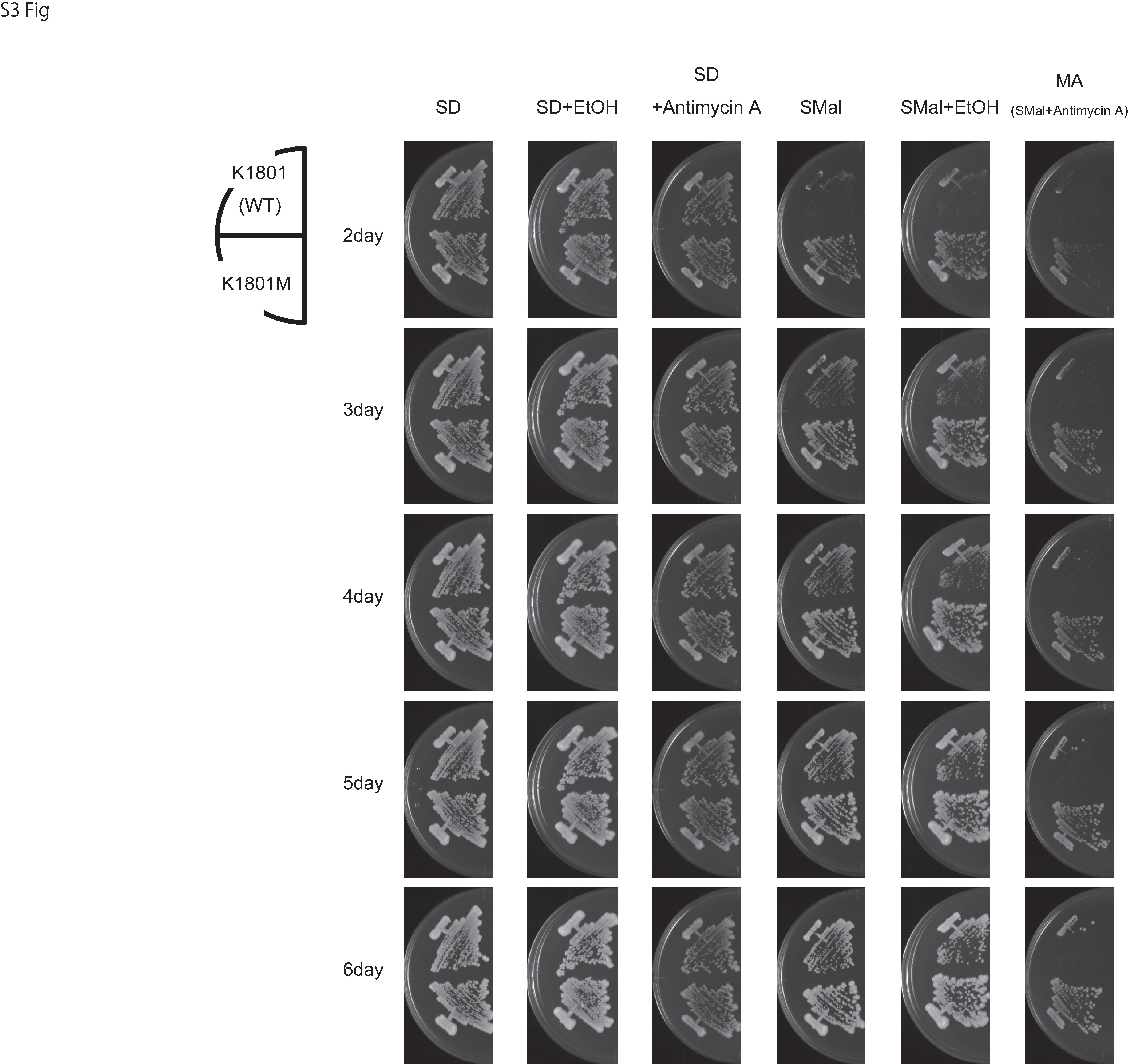

Supplement: S3 Fig — Each strain was streaked on SD, SD+EtOH (vehicle control), SD+Antimycin A, SMal, SMal+EtOH (vehicle control) and MA (SMal+antimycin A) plates. Each plate was incubated at 30°C, and growth was monitored daily over the course of 2–6 days. (TIF) [file pone.0198744.s003.tif]

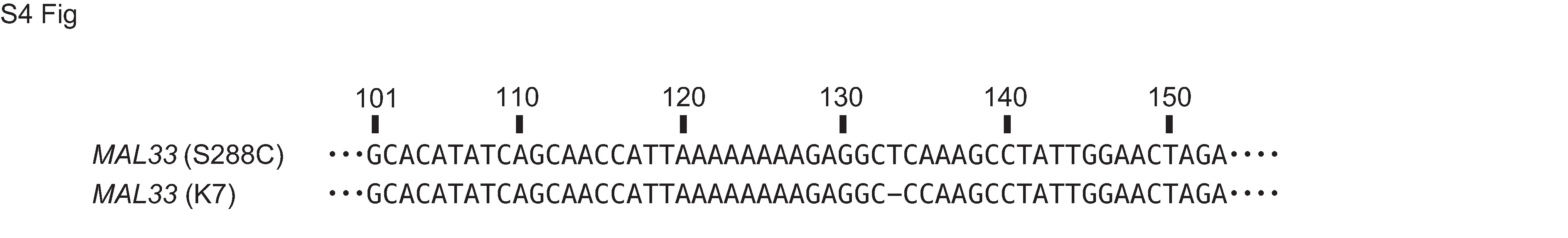

Supplement: S4 Fig — The MAL33 (S288C) sequence was downloaded from the Saccharomyces genome database (http://www.yeastgenome.org/), and the MAL33 (K7) sequence was downloaded from the DOGAN–Saccharomyces cerevisiae K7 database (http://www.bio.nite.go.jp/dogan/project/view/SC1) (TIF) [file pone.0198744.s004.tif]

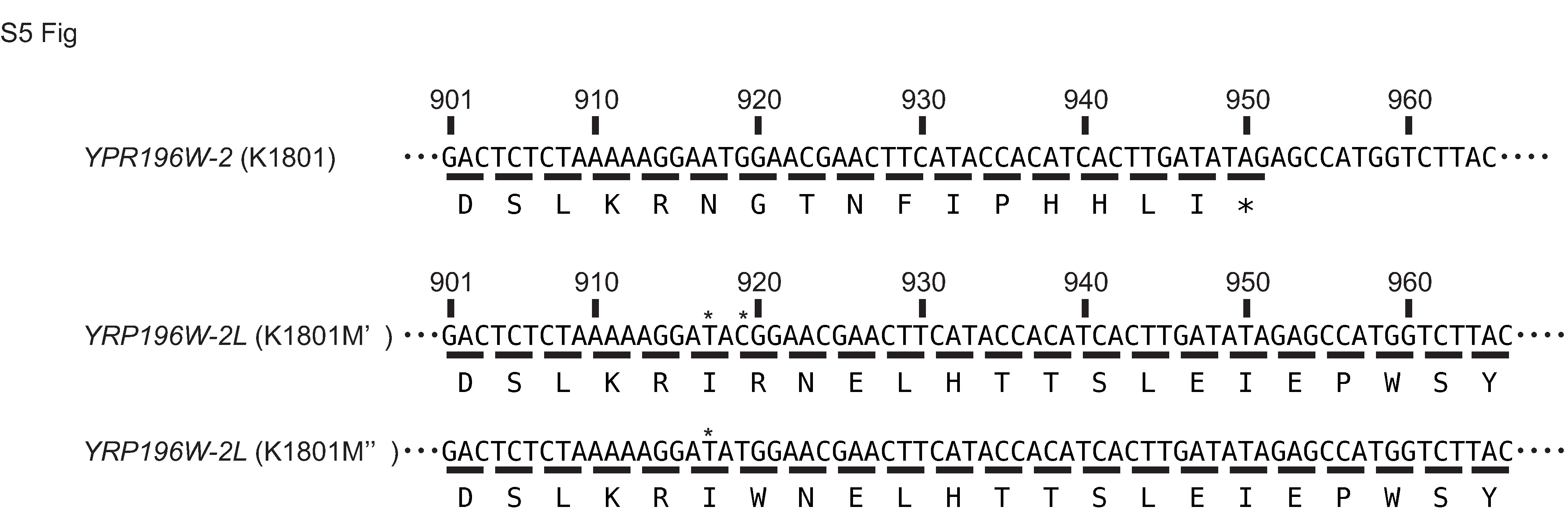

Supplement: S5 Fig — DNA (upper line) and amino acid (bottom line) sequences of YPR196W-2 homologs from other maltose-assimilative K1801-derived strains (K1801M’ and K1801M”) that were isolated through the mutagenesis screen of K1801 described in the Materials and Methods section. An asterisk is used to indicate the inserted or changed nucleotide in each strain. (TIF) [file pone.0198744.s005.tif]

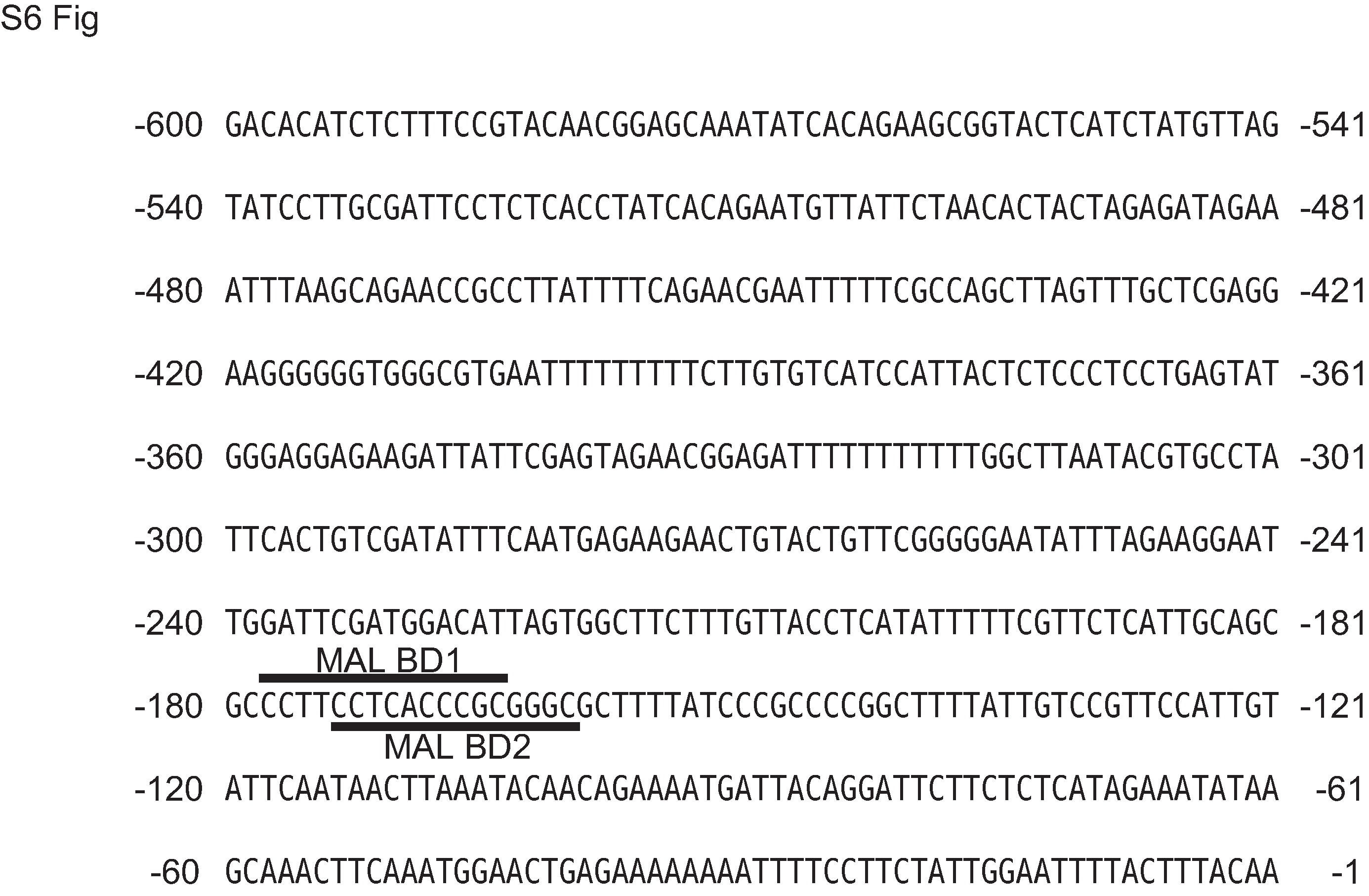

Supplement: S6 Fig — MAL-activator binding sites are indicated as MAL BD1 and MAL BD2. The consensus sequence of the MAL-activator binding site is MGCNNNNNNNNNMGS, where M is adenine or cytosine, S is guanine or cytosine, and N is any of the four deoxyribonucleotides. (TIF) [file pone.0198744.s006.tif]

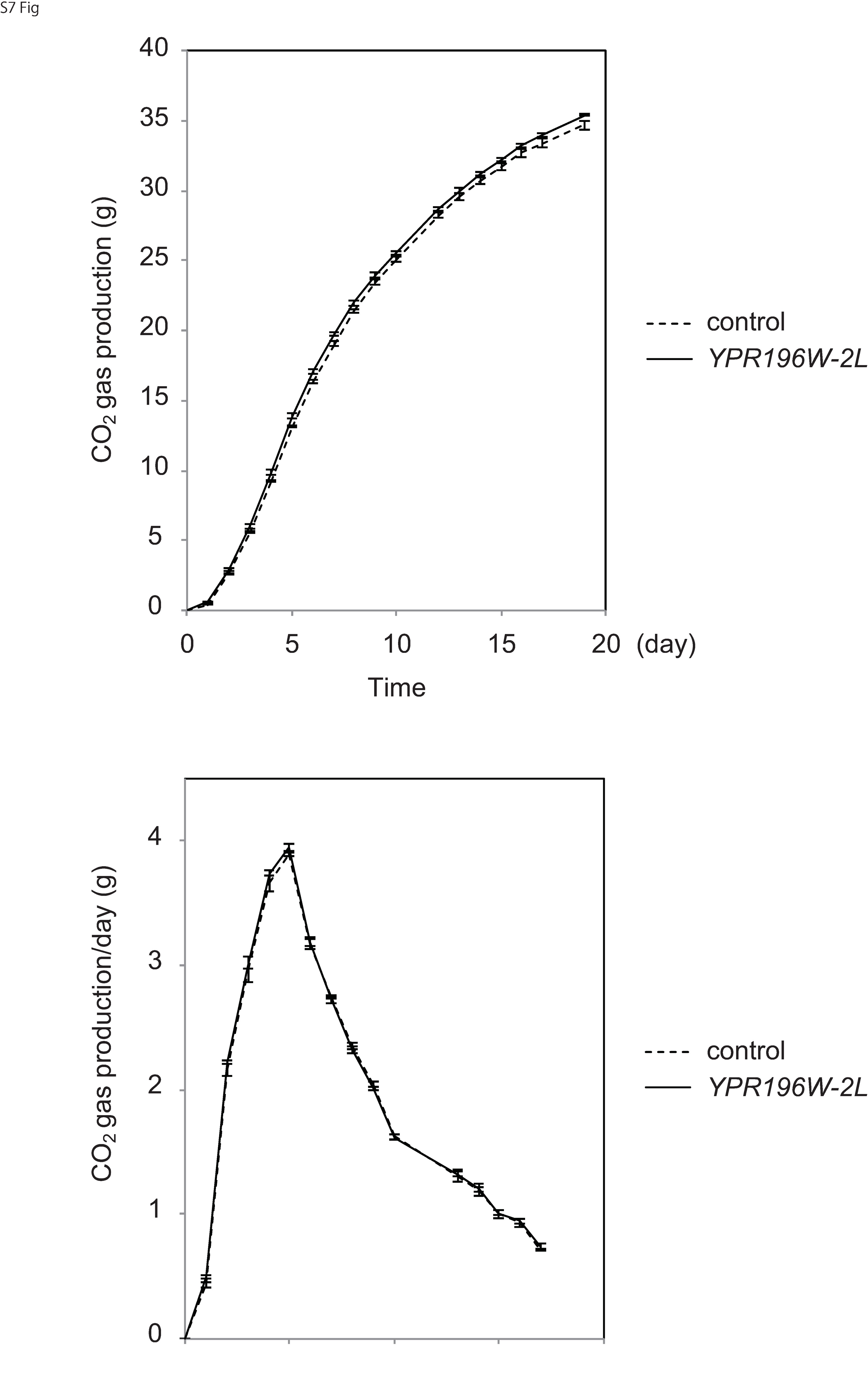

Supplement: S7 Fig — Sake mash containing yeast cells with control vector or YPR196W-2L plasmid were prepared in triplicate as described in the Materials and Methods section. Weight loss of the mash (reflecting CO2 gas emission) was measured at the same time each day. The upper panel shows total cumulative CO2 gas emission, and the bottom panel shows CO2 gas emission per day. Data are presented as the mean and standard deviation from three independent experiments. (TIF) [file pone.0198744.s007.tif]
