# Supplementary material for: MAL73, a novel regulator of maltose fermentation, is functionally impaired by single nucleotide polymorphism in sake brewing yeast
Source: PLoS One. 2018 Jun 12;13(6):e0198744. doi: 10.1371/journal.pone.0198744 (PMC5997316; doi:10.1371/journal.pone.0198744)
Supplement: S1 Table — (DOCX) [file pone.0198744.s008.docx]

|  |  | K1801+vector | K1801+*MAL73L* |
| --- | --- | --- | --- |
| Ethanol (%) |  | 18.8±0.3 | 18.7±0.6 |
| Specific gravity |  | 1.0021±0.0001 | 1.0014±0.0001 |
| *Sake* meter value |  | -3.07±0.82 | -1.97±0.29 |
| Acidity |  | 3.33±0.07 | 3.44±0.02 |
| Amino acidity |  | 2.13±0.04 | 2.04±0.04 |
| Concentration (ppm)　of | Phosphoric | 385.6±2.7 | 379.4±5.3 |
| Organic acids | Citric | 81.1±.057 | 81.5±1.5 |
|  | Pyruvic | n.d. | n.d. |
|  | Malic | 163.9±2.8 | 159.4±1.6 |
|  | Succinic | 559.4±3.9 | 556.1±0.7 |
|  | Lactic | 529.1±10.7 | 504.4±4.95 |
|  | Fumaric | n.d. | n.d. |
|  | Acetic | 232.1±5.2 | 234.6±11.4 |
| Volatiles | Ethyl acetate | 88.4±4.84 | 85.35±2.5 |
|  | n-Propanol | 172.0±1.7 | 170.0±2.2 |
|  | isoButanol | 64.6±0.5 | 63.5±1.2 |
|  | isoAmyl acetate | 4.84±0.2 | 4.66±0.1 |
|  | isoAmylalcohol | 187.3±1.5 | 186.5±1.0 |
|  | Ethyl Caproate | 2.90±0.08 | 2.95±0.10 |

Supporting table 1
